# Antimicrobial Resistance Prediction for Gram-Negative Bacteria via Game Theory-Based Feature Evaluation

**DOI:** 10.1038/s41598-019-50686-z

**Published:** 2019-10-09

**Authors:** Abu Sayed Chowdhury, Douglas R. Call, Shira L. Broschat

**Affiliations:** 1 School of Electrical Engineering and Computer Science, Washington State University, P.O. Box 642752 Pullman, Washington USA; 2 Paul G. Allen School for Global Animal Health, Washington State University, P.O. Box 647090 Pullman, Washington USA; 3 Department of Veterinary Microbiology and Pathology, Washington State University, P.O. Box 647040 Pullman, Washington USA

**Keywords:** Bioinformatics, Machine learning, Applied microbiology, Computational science, Computer science

## Abstract

The increasing prevalence of antimicrobial-resistant bacteria drives the need for advanced methods to identify antimicrobial-resistance (AMR) genes in bacterial pathogens. With the availability of whole genome sequences, best-hit methods can be used to identify AMR genes by differentiating unknown sequences with known AMR sequences in existing online repositories. Nevertheless, these methods may not perform well when identifying resistance genes with sequences having low sequence identity with known sequences. We present a machine learning approach that uses protein sequences, with sequence identity ranging between 10% and 90%, as an alternative to conventional DNA sequence alignment-based approaches to identify putative AMR genes in Gram-negative bacteria. By using game theory to choose which protein characteristics to use in our machine learning model, we can predict AMR protein sequences for Gram-negative bacteria with an accuracy ranging from 93% to 99%. In order to obtain similar classification results, identity thresholds as low as 53% were required when using BLASTp.

## Introduction

Bacteria can cause bloodstream infections, and with the increasing prevalence of antimicrobial resistance (AMR) in bacteria treatment can become complicated^1–7^. AMR results in increased mortality and an increase in the duration of hospitalization. Every year, millions of people in the United States are infected by AMR bacteria, and thousands of people die^8,9^. Hence, accurate identification of AMR in bacteria is essential for the proper administration of appropriate antibacterial agents. To detect AMR in bacteria, *in vitro* cultures are used to monitor the growth of bacteria for different concentrations of drugs and may require several days to obtain accurate antibiotic susceptibility results^10^. In addition, many bacteria cannot be cultured, and a large number of these are becoming available via metagenomic studies^11,12^.

With breakthroughs in whole genome sequencing (WGS) method, it is possible to apply sequence alignment approaches such as best-hit methods to identify AMR genes using sequence similarity in public databases^4,13,14^. These methods show good performance in identifying known and highly conserved AMR genes and produce small number of false positives, *i*.*e*., detecting non-AMR genes as AMR genes^15^. However, they may not be able to to find AMR sequences that have high dissimilarity with known AMR genes, producing unacceptable numbers of false negatives^13,16,17^. A machine learning approach can be used as an alternative solution for identifying putative AMR genes. To train a machine learning algorithm to detect AMR sequences, training data are needed in the form of protein sequences for known AMR genes (positive training data) and protein sequences that are known not to be AMR genes (negative training data). From these training data, we must determine what protein characteristics distinguish AMR genes from non-AMR genes. These characteristics are known as features. Numerous features exist for protein sequences, and a goal of any accurate machine learning model is to determine which features provide the most useful information. Recently, two studies have proposed machine learning approaches for predicting AMR genes. Arango-Argoty *et al*. discuss a deep learning approach—DeepARG^18^ to identify novel antimicrobial resistance genes from metagenomic data. DeepARG employs an artificial neural network based classifier, taking into account the similarity distribution of sequences to all known AMR genes. The other approach is the pairwise comparative modelling (PCM)^19^ which leverages protein structure information for AMR sequence identification. PCM builds two structural models for each candidate sequence with respect to AMR and non-AMR sequences, and a machine learning model is applied to find the best structural model for determining whether the sequence belongs to an AMR or non-AMR family. In contrast to these earlier works, we consider all possible candidate features for protein sequences based on the composition, physicochemical, evolutionary, and structural characteristics of protein sequences whose sequence identity ranges between 10% and 90%.

The earlier works discussed above did not apply any feature reduction strategy to find the most relevant, non-redundant and interdependent features. Identifying important features from a set of features to attain high classification accuracy is a challenging problem in machine learning because irrelevant or redundant features can compromise accuracy. Several feature selection techniques can be used to obtain an optimal feature set, and these are broadly classified into three categories: embedded, wrapper, and filter approaches. Both embedded and wrapper methods^20–22^ are tightly coupled with a particular learning algorithm, and both achieve good classification accuracy. However, these approaches have a high computational overhead and less generalization of features. Alternatively, filter methods measure feature relevance by considering the intrinsic properties of the data^23–25^. In addition, the filter approach has a lower computational cost than the other methods, and it facilitates comparable accuracy for most classifiers^26^. Thus, we only considered filter method for our feature selection approach.

Most filter methods reject features that are poorly predictive when used alone, even though they may work well when combined with other variables^26,27^. In contrast, in this paper we introduce a game theoretic dynamic weighting based feature evaluation (GTDWFE) approach in which features are selected one at a time based on relevance and redundancy measurements with dynamic re-weighting of candidate features based on their interdependency with the current selected features. Re-weighting is determined using a Banzhaf power index^28^, and features are not necessarily rejected because they are poorly predictive as single variables. Instead we consider how features work together as a whole using a game theory approach. In simple terms, game theory is the study of mathematical models for determining how the behavior of one participant depends on the behavior of other participants. We consider features from the protein sequences—both AMR and non-AMR—of the Gram-negative bacterial genera *Acinetobacter*, *Klebsiella*, *Campylobacter*, *Salmonella*, and *Escherichia* for acetyltransferase (*aac*), *β*-lactamase (*bla*), and dihydrofolate reductase (*dfr*). Next we apply our game theory approach to select a small subset of features from the bacterial protein sequences, and finally we utilize this small feature subset to predict AMR genes using a support vector machine (SVM)^29^. We use protein sequences from different Gram-negative bacteria *Pseudomonas* spp., *Vibrio* spp., and *Enterobacter* spp. to test our classifier. We also make a performance comparison between our classifier and BLASTp.

## Results

### Interdependent group size based comparative analysis

We compared the classification performance of our method using an SVM with an interdependent group size of $\delta \in [1,3]$ where *δ* is used in the computation of the Banzhaf power index. Using the top *k* features and 30 different feature subsets ($1\le k\le 30$) of *Acinetobacter*, *Klebsiella*, *Campylobacter*, *Salmonella*, and *Escherichia* and dividing the dataset into 70%/30% training/test samples, we tuned the SVM based on an equal number of positive and negative samples, to determine the best parameters for the SVM models. We selected the radial basis function kernel for each SVM^30,31^ but different *C* and *γ* values for each feature subset. *C* is used to control the cost of misclassification in the SVM, and *γ* is the kernel parameter. We used the resulting models trained using 70% of the dataset to identify putative AMR genes for the remaining data set. The numbers of the best feature subsets using oversampling and undersampling techniques for different *δ* values are shown in Table 1 where the classification accuracy achieved for each respective best feature subset is shown in parentheses. Oversampling and undersampling are methods in data analytics for balancing sets of data for which there are inherently more samples of one class than another. For this work, the number of positive samples (AMR) is smaller than the number of negative samples (non-AMR). To compensate, we duplicated the positive training samples (oversampling) and we removed some of the negative training samples (undersampling) to achieve balanced datasets.

**Table 1:** Classification performance for different *δ* values (corresponding classification accuracy in parentheses).

AMR	Oversampling			Undersampling		
	*δ* = 1	*δ* = 2	*δ* = 3	*δ* = 1	*δ* = 2	*δ* = 3
acetyltransferase (*aac*)	6 (0.97)	6 (0.97)	6 (0.97)	5 (0.97)	5 (0.97)	5 (0.97)
*β*-lactamase (*bla*)	15 (1)	19 (1)	18 (1)	9 (0.97)	9 (0.97)	11 (0.97)
dihydrofolate reductase (*dfr*)	5 (1)	5 (1)	5 (1)	18 (0.96)	28 (1)	25 (1)

The classification accuracies achieved for *aac*, *bla*, and *dfr* vary from 96% to 100%. We also determined $\delta =3$ to be the overall best interdependent group size, so we set *δ* to 3 for the remainder of our analyses. The *C* and *γ* parameter values for the SVM radial model for selecting each best feature subset with $\delta =3$ are listed as a supplementary table (Table S1).

### Feature selection method comparative analysis

**Figure 1:**
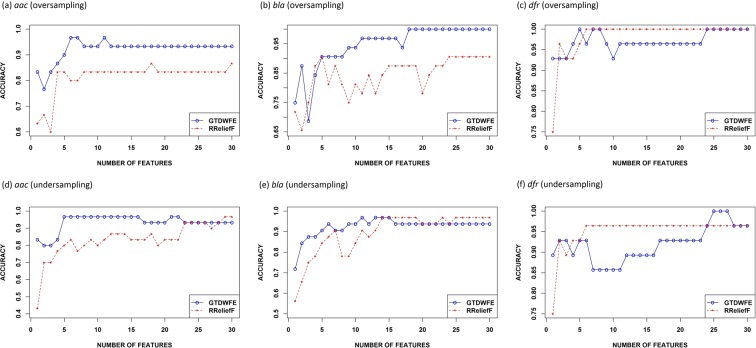
Comparison between GTDWFE and RReliefF accuracies for oversampling and undersampling. Accuracies are given as a function of the number of features used.

To assess the performance of our GTDWFE feature evaluation method, we compared it with a popular feature selection algorithm — RReliefF^32^. RReliefF is an updated version of Relief and ReliefF^33,34^, a filter-based method that uses distance between instances to find the relevance weight of each feature to rank the features. For RReliefF, we considered 5 neighbors and 30 instances as suggested in a previous study^35^. We used $\delta =3$ in our feature selection approach. Comparisons of the results for the two methods are shown in Fig. 1 for oversampling and undersampling. As can be seen in these results, the maximum accuracies our GTDWFE achieved *w*.*r*.*t*. the number of features are better than those of RReliefF for all cases. Note that in some cases, RReliefF achieved equal maximum accuracies as GTDWFE method, but the latter required fewer number of features.

### Identification of antimicrobial-resistance proteins from *Pseudomonas*, *Vibrio*, and *Enterobacter*

**Figure 2:**
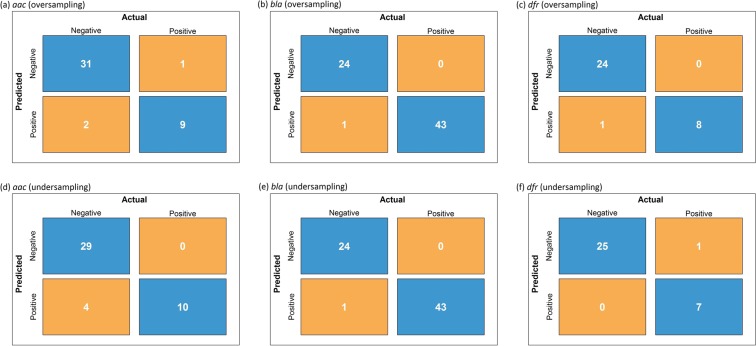
Confusion matrices for oversampling and undersampling.

To acquire a better understanding of whether the GTDWFE method can identify putative AMR genes in Gram-negative bacteria, we trained SVM classifiers using 100% of the positive and negative samples for *Acinetobacter*, *Klebsiella*, *Campylobacter*, *Salmonella*, and *Escherichia*. We used the same features obtained previously for $\delta =3$ to train the SVM model. We then tested the SVM classifiers using the same features for positive and negative samples for *Pseudomonas*, *Vibrio*, and *Enterobacter*. Importantly, for acetyltransferase, we used eight non-AMR samples of acetyltransferase to ascertain whether the SVM classifier was able to distinguish between resistant and non-resistant samples of acetyltransferase. The confusion matrices for both oversampling and undersampling cases are shown in Fig. 2. In a confusion matrix, ‘Positive’ and ‘Negative’ indicate AMR (positive) and non-AMR (negative) classes, respectively, and falling diagonal entries indicate correctly identified instances. For oversampling, the GTDWFE method achieved accuracies of 0.93, 0.99, and 0.97 for *aac*, *bla*, and *dfr*, respectively. Moreover, 6 of the 8 non-AMR samples of acetyltransferase were correctly predicted to be negative. For undersampling, the GTDWFE approach had accuracies of 0.91, 0.99, and 0.97 for *aac*, *bla*, and *dfr*, respectively. Of the 8 non-AMR samples, 5 were correctly predicted to be negative samples. Here, as for our test cases in the previous section, the GTDWFE method gives better results when oversampling is used.

**Figure 3:**
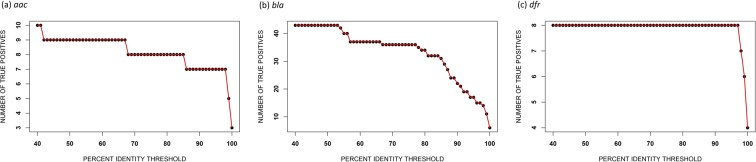
Identification of AMR sequences in *Pseudomonas*, *Vibrio*, or *Enterobacter* using BLASTp as a function of percent identity using AMR sequences from *Acinetobacter*, *Klebsiella*, *Campylobacter*, *Salmonella*, and *Escherichia*.

Based on the results above, we conclude that our game theory approach for determining protein features for use in a machine learning algorithm can be used with high accuracy to predict AMR in Gram-negative bacteria. We used training data from five different Gram-negative bacterial genera and predicted AMR in three different Gram-negative bacterial genera. The results based on oversampling were the most accurate with 93% accuracy for acetyltransferase resistance, 97% accuracy for dihydrofolate reductase resistance, and 99% accuracy for *β*-lactamase resistance. The performance of our game theory method was also compared with the BLASTp results considering default parameter settings (https://blast.ncbi.nlm.nih.gov/Blast.cgi?PAGE=Proteins). The outcomes shown in Fig. 3 are the number of matched AMR protein sequences as a function of percent identity for *Pseudomonas*, *Vibrio*, and *Enterobacter* using the AMR genes from *Acinetobacter*, *Klebsiella*, *Campylobacter*, *Salmonella*, and *Escherichia*. For example, considering percent identity ≥90 for a sequence from *Pseudomonas*, *Vibrio*, or *Enterobacter* to be matched (true positive) with an AMR sequence from *Acinetobacter*, *Klebsiella*, *Campylobacter*, *Salmonella*, or *Escherichia*, we obtain eight true positives out of ten *aac* sequences, 22 true positives out of 43 *bla* sequences, and eight true positives out of eight *dfr* sequences. The results for *dfr* are much better, but this may in part be due to the limited overall diversity of available *dfr* sequences. Note that the percent identity threshold has to be as low as 41% to obtain accurate results for *aac* and *bla*, but this results in an increased number of false positives. As an example, when the percent identity threshold is set to 41%, six out of eight histone acetyltransferases are incorrectly detected as AMR samples, indicating a large false positive rate. Therefore, using an appropriate threshold setting for BLASTp compromises the accuracy of the results. To obtain the equal true positives as for our oversampling cases (Fig. 2), the percent identity threshold of BLASTp need to be 67% for *aac* and 53% for *bla*; however, these sequence identity thresholds produce three false positives for *aac* (two of them are histone acetyltransferases) and six false positives for *bla*. Thus, false positive rates using BLASTp classification for *aac* and *bla* are still high compared to our GTDWFE algorithm. Our machine learning approach provides greater accuracy than conventional methods for highly diverse protein sequences.

## Discussion

In this paper we presented a machine learning method for prediction of antimicrobial-resistance genes for three antibiotic classes. The strength of a machine learning algorithm is that it uses features based on the structural, physicochemical, evolutionary, and compositional properties of protein sequences rather than simply their sequence similarity. The novel game theory approach we used to determine protein features for our machine learning algorithm has not been used previously for such a purpose and is especially powerful because features are chosen on the basis of how well they work together as a whole to identify putative antimicrobial-resistance genes by taking into account both the relevance and interdependency of features. As such, we were able to use protein sequences to train the machine learning algorithm using functionally-equivalent amino acid sequences with shared identity that ranged from 10% to 90%. The algorithm was then able to correctly identify genes from an independent data set with 93% to 99% accuracy. The only way this can be achieved by means of a best-hit approach such as BLASTp is by considering sequence matches with as low as 53% similarity. Compared to our approach, this then leads to a greater number of false positives, that is, sequences incorrectly identified as antimicrobial-resistance genes.

Our work included collection of resistance and non-resistance protein sequences, feature extraction, feature evaluation for dimension reduction, handling of imbalanced data sets, and comparison of our method with an existing feature selection approach. The RReliefF algorithm for selecting features is well-known for its accuracy and ability to rank features by their importance, but it does not account for feature interdependence. This was made clear by comparison between results obtained using the game theory algorithm, GTDWFE, and RReliefF. GTDWFE achieved the highest accuracy for all cases using fewer features than RReliefF because of its reduction in irrelevant and/or redundant information. The results of our approach using oversampling were better than those using undersampling.

With growth in both antimicrobial resistance and the number of sequenced genomes available, implementation of machine learning models for accurate prediction of AMR genes represents a significant development toward new and more accurate tools in the field of predictive antimicrobial resistance. In future work, we will create a user-friendly and publicly available program for predicting AMR in bacteria based on the method presented in this paper.

## Methods

### Data collection

Amino acid sequences for antimicrobial-resistance genes were retrieved from the Antibiotic Resistance Genes Database (ARDB)^36^, and non-AMR sequences were obtained from the Pathosystems Resource Integration Center (PATRIC)^37^. A BLASTp search using default parameter settings was performed to find all matching sequences. Initial AMR sequences for the Gram-negative bacteria *Acinetobacter* spp., *Klebsiella* spp., *Campylobacter*spp., *Salmonella* spp., and *Escherichia* spp. numbered 387 for *aac*, 1113 for *bla*, and 804 for *dfr*; there were 159 non-AMR sequences (73 essential genes and 86 histone acetyltransferesases^38^) randomly chosen. Because of the number of duplicate sequences, we used CD-HIT^39,40^ to find the unique sequences. Sequences having ≥90% similarity were removed for further consideration. The final number of unique sequences obtained were 33 *aac*, 43 *bla*, and 28 *dfr* AMR sequences and 71 non-AMR sequences (64 essential genes and 7 histone acetyltransferases). This data set was used as the training and test set for our model. The histone acetyltransferases together with the essential sequences were used as negative training data only for the *aac* classifier. For *bla* and *dfr*, only essential sequences were used as negative training data. In addition to the training/test data set, 199 *aac*, 588 *bla*, 66 *dfr* AMR sequences and 82 non-AMR sequences (35 essential genes and 47 histone acetyltransferases) for the Gram-negative bacteria *Pseudomonas* spp., *Vibrio* spp., and *Enterobacter* spp. were collected from the data sources indicated above. After application of CD-HIT, 10 *aac*, 43 *bla*, and 8 *dfr* AMR sequences and 33 non-AMR sequences (25 essential genes and 8 histone acetyltransferases) were retained. These were used to test the accuracy of the final classifier. Again, histone acetyltransferases were used only in the *aac* model. Note that sequence similarity for the AMR sequences could be quite low.

### Protein features

A literature search was used to identify the composition, physicochemical characteristics, and secondary structure properties of protein sequences^41–46^. As a result of this search, we created 20*D* feature vectors based on amino acid composition with each of the 20 feature values in a vector representing one of the 20 amino acids. Next the composition, transition, and distribution (CTD) model proposed by Dubchak *et al*.^47,48^ was used to retrieve global physicochemical features from protein sequences. The CTD model results in a 3*D* feature vector for composition, a 3*D* feature vector for transition, and a 15*D* feature vector for distribution. As there 8 physicochemical amino acid properties, the CTD paradigm provides a total of $(3+3+15)\times 8=168$ features. We obtained evolutionary-relevant features using a position-specific scoring matrix (PSSM). After producing a PSSM for a protein sequence by applying PSI-BLAST^49^, we computed transition scores between adjacent amino acids which resulted in a 400*D* feature vector for each protein sequence.

Finally, features were obtained for the secondary structure of proteins which provides relevant information in protein fold recognition. PSIPRED^50^ was applied to sequences to predict their secondary structures. These were used as described in previous studies^43,44,51–53^ to obtain our secondary structure features. Location-oriented features were produced from the spatial arrangements of the *α*-helix, *β*-strand, *γ*-coil states. Normalized maximum spatially consecutive states in the secondary structure sequences were also calculated. Additionally, we retrieved features from segment sequences by disregarding the coil portions in the secondary structure. In such a way, a total of six features were generated from the protein structure information.

Three global information features were generated from the structure probability matrix (SPM) produced by PSIPRED. Local information features were acquired by dividing the SPM into *δ* submatrices, each with $\lfloor \tfrac{n}{\delta }\rfloor \times 3$ entries. By selecting $\delta =8$, we generated 3*D* features for a particular sub-matrix using the same approach considered for the generation of global information features. Hence, we obtained 3 × 8 = 24 local information features. In total, 3 + 24 = 27 features with global and local information were generated.

By combining all the features, we obtained a 621*D* high-dimensional feature vector. Detailed descriptions of all the extracted candidate features together with the formulas used to calculate values can be found in our previous work^54^. The objective of this work was to then reduce the dimension of our feature vector in such a way as to produce accurate machine learning predictions for AMR.

### Feature evaluation

We adopted a feature evaluation method to select a small number of features based on a relevance, redundancy, and interdependency estimation of all the features. In our approach, we calculated the weight of a feature based on the features selected earlier, and weight readjustment of a feature was performed dynamically when a new selected feature was added to the previously selected feature subset. Here, the weight of a feature actually resembles the interdependence relationship with features previously selected. The details of our feature selection approach called the game theoretic dynamic weighting based feature evaluation (GTDWFE) algorithm are presented in Algorithm 1.

**Algorithm 1:**
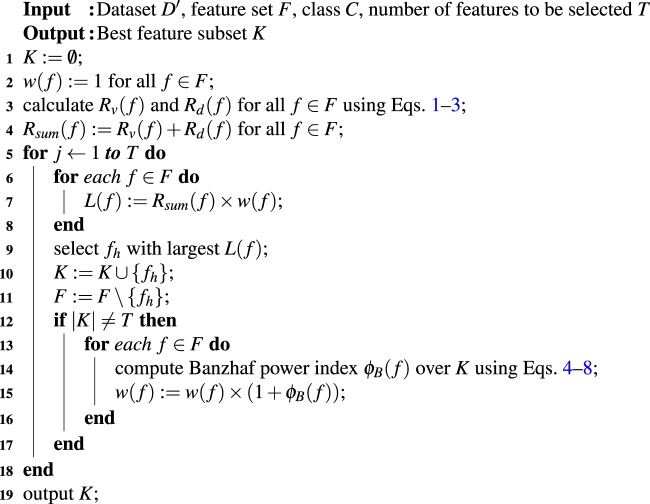
Game theoretic dynamic weighting based feature evaluation (GTDWFE) algorithm.

Algorithm 1 takes data set *D*′, feature set *F*, binary classes *C*, and number of features to be selected *T* and outputs the best feature subset *K*. To implement this algorithm we first initialize the parameters, setting the same weight to each feature, *i*.*e*., weight *w*(*f*) of a feature *f* is set to 1 initially (line 2). Relevance to the target class *R*_*v*_(*f*) and similarity value of a feature *R*_*d*_(*f*) are calculated for all features (line 3). The greater the relevance of a feature to the target (AMR or non-AMR sequence), the more it can contribute to the prediction by sharing information with the target class. Also, the greater the distance of a feature from all other features, the lower the similarity of the feature with the remaining features, which indicates lower redundancy. We computed Pearson’s correlation coefficient between a feature and class using Eq. 1, that is, we estimated the linear correlation between feature *f* and class *C*. Pearson’s correlation coefficient (denoted by $\rho$) is computed using

1 $${R}_{v}(f)=\rho =\frac{{\bf{E}}[(f-{\mu }_{f})\,(C-{\mu }_{C})]}{{\sigma }_{f}{\sigma }_{C}}$$

where the expectation is represented by **E**, *μ*_*f*_ and *μ*_*c*_ are the means, and *σ*_*f*_ and *σ*_*c*_ correspond to the standard deviations for *f* and *c*, respectively. We used the absolute value $|\rho |$ as the value of *R*_*v*_(*f*) for the feature *f*. To find the *R*_*d*_(*f*) value of a feature *f*, we measured the average distance of a feature *f* with all other features using the Tanimoto Coefficient $TC(f,{f}_{j})$ given in Eqs. 2 and 3. Here, *d* is the total number of features, for our case $d=621$.

2 $${R}_{d}(f)=\frac{1}{d-1}\,{\sum }^{}\,TC(f,{f}_{j}),1\le j\le d,f\ne {f}_{j}$$

3 $$TC(f,{f}_{j})=\frac{f.{f}_{j}}{\parallel \,f\,{\parallel }^{2}+\parallel \,{f}_{j}{\parallel }^{2}-f.{f}_{j}}$$

After summing the *R*_*v*_( *f* ) and *R*_*d*_(* f* ) values for all features (line 4), the algorithm iterates until the required *T* features have been selected. For every iteration the value of *L*( *f* ) is computed (lines 6–8), and the feature with the largest *L*( *f* ) is selected, added into subset *K*, and then eliminated from feature set *F* (lines 9–11).

The weights of the remaining candidate features are recalculated in each iteration to determine the impact of the candidate features on the features selected earlier (lines 13–16). We used the Banzhaf power index^28^ to readjust the weight *w*( *f* ) of a feature *f*. The Banzhaf power index is widely used in game theory approaches to measure the power of a player to form a coalition with a set of other players *S*. Winning and losing coalitions in a game are those coalitions with $v(S)=1$ and $v(S)=0$, respectively. For every winning coalition of $S\cup \{r\}$ if *S* would lose without player *r*, then *r* is crucial to winning the game. Because player *r* is a feature, we made a slight modification to the original Banzhaf power index, and the updated definition of the Banzhaf power index for a player *r* is given in Eq. 4.

4 $${\varphi }_{B}(r)=\frac{1}{|{\Pi }_{\delta }|}\,\sum _{S\subseteq {\Pi }_{\delta }}\,{\Delta }_{r}(S)$$

where the marginal contribution of the feature *r* to all coalitions is ${\Delta }_{r}(S)$ where ${\Delta }_{r}(S)=v(S\cup r)-v(S)$. *δ* is the upper bound of the cardinality of *S*, and $|{\Pi }_{\delta }|$ gives the total number of subsets of *F*\*r* bounded by *δ*. This means that $\{{\Pi }_{g}\}\in S$, and *g* is the cardinality of a feature subset with $g=1,2,\ldots ,\delta$.

If we consider two features *r* and *t* as two players, we can calculate their interdependence using Eq. 5 where *C* represents the binary classes 1 (AMR or positive class) and −1 (non-AMR or negative class).

5 $$\tau (r,t)=\{\begin{array}{ll}1 & {\rm{if}}\,I({f}_{t};C|{f}_{r}) > I({f}_{t};C)\\ 0 & {\rm{otherwise}}\end{array}$$

We can formulate ${\Delta }_{r}(S)$ as

6 $${\Delta }_{r}(S)=\{\begin{array}{ll}1 & {\rm{if}}\,I(S;C|{f}_{r})\ge 0,{\sum }_{{f}_{t}\in S}\,\tau (r,t)\ge \frac{|S|}{2}\\ 0 & {\rm{otherwise}}\end{array}$$

From these equations we see that a feature is important if it increases the relevance of the subset *S* with the binary classes (*i*.*e*., 1 and −1), and it should be interdependent with 50% or more of the members. The *I*’s in these equations are the mutual information and conditional mutual information and are calculated using Eqs. 7 and 8, respectively, where *U*, *V*, and *Z* are random variables.

7 $$I(U;V)=\sum _{u\in U}\,\sum _{v\in V}\,p(u,v)\,\log \,\frac{p(u,v)}{p(u)p(v)}$$

8 $$I(U;V|Z)=\sum _{u\in U}\,\sum _{v\in V}\,\sum _{z\in Z}\,p(u,v,z)\,\log \,\frac{p(u,v|z)}{p(u|z)p(v|z)}$$

The algorithm is aborted when *T* features have been selected from the feature set *F*. The output feature subset *K* is the optimal feature subset for providing maximum relevance, minimum redundancy, and informative interdependence relations (line 19).

### Imbalanced data

An imbalanced data set has significantly more of one class of training data than the other. Such a data set leads a classifier to predict the majority class more accurately while lowering the accuracy of the minority class predictions. This happens because of over-training of the majority class and under-training of the minority class. To avoid this, a data set can be balanced via sampling techniques^55,56^. There are two major sampling methods, namely oversampling and undersampling. In oversampling, we duplicate data from the minority class to balance the data set. In undersampling, we remove data from the majority class to balance the data set. In this study, we applied both over-sampling and under-sampling techniques to measure the performance of our prediction model.

### Support Vector Machine

A Support Vector Machine (SVM)^29^ is a supervised machine learning algorithm that represents each data item as a point in *p*-dimensional space and constructs a hyperplane (decision boundary) to separate data points into two groups. The core set of vectors identifying the hyperplane are known as support vectors. Unlike many classifiers, an SVM avoids overfitting by regularizing its parameters. Overfitting occurs when a classifier models the training data so well that it affects the accuracy of the classifier on new data. The SVM has proven to be a good classifier for protein sequences, classifying them with high accuracy. As predicting AMR is a binary classification problem, we chose an SVM for this work. A radial basis function^30,31^ was used as its kernel.

### R Scripts and Packages

R (https://cran.r-project.org/mirrors.html), a popular programming language for statistical analysis, provides many built-in packages for data analysis and machine learning. We wrote scripts in R to implement our GTDWFE feature evaluation algorithm and SVM classifier. We utilized the R *stats* (v3.5.0) package to perform the Pearson’s correlation measurements, the *proxy* (v0.4–22) package for calculating the Tanimoto coefficients, and *infotheo* (v1.2.0) for finding the mutual information and conditional mutual information. We applied the ROSE (v0.0–3) package to balance the data set and the *tune()* function in the *e1071* (v1.6–8) package to find the best SVM parameters. The *caret* (v4.20) package was used to generate confusion matrices. Finally, we utilized the *FSelector* (v0.31) package to implement RReliefF^32^.

### Performance measurement

We measured the performance of the SVM classifier with our optimized feature set by generating confusion matrices which were used to calculate classification accuracies. Table 2 shows the structure of a confusion matrix, where *TP*, *TN*, *FP*, and *FN* are true positives (positives accurately classified), true negatives (negatives accurately classified), false positives (negatives classified as positives), and false negatives (positives classified as negatives), respectively. Classification accuracy is calculated from these values as given in Eq. 9.

9 $$Accuracy=\frac{TP+TN}{TP+TN+FP+FN}$$

**Table 2:** Confusion matrix for classification performance.

Actual / Predicted	Negative	Positive
Negative	TN	FN
Positive	FP	TP

### Accession codes

NCBI^57^ accession numbers for all proteins used in this work are listed in Supplementary Tables S2–S11.

## Supplementary information

Supplementary Tables

## Data Availability

The R scripts written to implement our method are available at https://github.com/abu034004/GTDWFE.

## Data Availability

All experimental data are available at https://github.com/abu034004/GTDWFE.
