# Publisher Correction: Antimicrobial Resistance Prediction for Gram-Negative Bacteria via Game Theory-Based Feature Evaluation

**DOI:** 10.1038/s41598-020-58759-0

**Published:** 2020-01-30

**Authors:** Abu Sayed Chowdhury, Douglas R. Call, Shira L. Broschat

**Affiliations:** 1 School of Electrical Engineering and Computer Science, Washington State University, P.O. Box 642752, Pullman, Washington USA; 2 Paul G. Allen School for Global Animal Health, Washington State University, P.O. Box 647090, Pullman, Washington USA; 3 Department of Veterinary Microbiology and Pathology, Washington State University, P.O. Box 647040, Pullman, Washington USA

Correction to: *Scientific Reports* 10.1038/s41598-019-50686-z, published online 09 October 2019

This Article contains errors in Table 2 where the reported table heading was incorrect. The correct heading should read “Predicted\Actual”.

