# Supplementary material for: Antimicrobial Resistance Prediction for Gram-Negative Bacteria via Game Theory-Based Feature Evaluation
Source: Sci Rep. 2019 Oct 9;9:14487. doi: 10.1038/s41598-019-50686-z (PMC6785542; doi:10.1038/s41598-019-50686-z)
Supplement: Supplementary file 1 — Supplementary Tables [file 41598_2019_50686_MOESM1_ESM.pdf]

Antimicrobial Resistance Prediction for Gram-Negative Bacteria via Game Theory-Based Feature Evaluation

Abu Sayed Chowdhury[1,*], Douglas R. Call[1,2], and Shira L. Broschat[1,2,3]

[1]School of Electrical Engineering and Computer Science, Washington State University, P.O. Box 642752, Pullman, Washington, USA

[2]Paul G. Allen School for Global Animal Health, Washington State University, P.O. Box 647090, Pullman, Washington, USA

[3]Department of Veterinary Microbiology and Pathology, Washington State University, P.O. Box 647040, Pullman, Washington, USA

Parameters used to tune SVM models

Table S1: C and γ values selected for the SVM radial model for $\delta = 3$ (corresponding best feature subset in parentheses).

AMR	Oversampling		Undersampling	
	C	γ	C	γ
acetyltransferase (*aac*)	8 (6)	0.16667 (6)	4 (5)	0.2 (5)
β-lactamase (*bla*)	4 (18)	0.0556 (18)	4 (11)	0.0909 (11)
dihydrofolate reductase (*dfr*)	4 (5)	0.2 (5)	4 (25)	0.04 (25)

Unique protein names with NCBI accession numbers obtained from *Acinetobacter*, *Klebsiella*, *Campylobacter*, *Salmonella*, and *Escherichia*

Table S2: List of 33 sequences that convey resistance to *aac*.

NCBI accession number	Protein name
ESD46483	aminoglycoside 3-N-acetyltransferase
SLR23795	aminoglycoside acetyltransferase
ALP55389	aminoglycoside N3'-acetyltransferase (plasmid)
KYH57362	aminoglycoside 3-N-acetyltransferase
KDJ63161	aminoglycoside N(3')-acetyltransferase IV
KQD27496	gentamicin 3'-acetyltransferase
WP_015243635	AAC(3)-I family aminoglycoside N-acetyltransferase
RFN37427	Aminoglycoside N(3')-acetyltransferase III
EHC71407	Aminoglycoside acetyltransferase
WP_016541245	MULTISPECIES: aminoglycoside N-acetyltransferase AAC(6')-Ih
WP_005202238	MULTISPECIES: AAC(6')-Ighjkrstuvwx family aminoglycoside N-acetyltransferase
WP_005145021	AAC(6')-Ighjkrstuvwx family aminoglycoside N-acetyltransferase
WP_005288246	MULTISPECIES: AAC(6')-Ighjkrstuvwx family aminoglycoside N-acetyltransferase
WP_005269797	AAC(6')-Ighjkrstuvwx family aminoglycoside N-acetyltransferase
WP_004670361	AAC(6')-Ighjkrstuvwx family aminoglycoside N-acetyltransferase
WP_017395772	AAC(6')-Ighjkrstuvwx family aminoglycoside N-acetyltransferase

WP_005062866	AAC(6')-Ighjkrstuvwx family aminoglycoside N-acetyltransferase
WP_085334908	aminoglycoside N-acetyltransferase AAC(6')-Id
WP_069722139	cryptic aminoglycoside N-acetyltransferase AAC(6')-Iy/Iaa
OKB62973	AacA4 family aminoglycoside N(6')-acetyltransferase
CAG26810	6'-N-aminoglycoside acetyltransferase type IV
ABN48510	AacA4
KMH34956	aminoglycoside N(6')-acetyltransferase type 1
BAM45412	aminoglycoside-(6')-N-acetyltransferase type Ib
AFG73657	aminoglycoside acetyltransferase
WP_012477386	aminoglycoside 6'-N-acetyltransferase AacA38
ANS91765	Aminoglycoside N(6')-acetyltransferase type 1 (plasmid)
ADX02779	Aminoglycoside 6'-N-acetyltransferase
YP_006953604	aminoglycoside acetyltransferase
WP_024437351	AAC(6')-Ia family aminoglycoside 6'-N-acetyltransferase AacA43
WP_032489895	aminoglycoside 6'-N-acetyltransferase AAC(6')-Ia
YP_001552089	aminoglycoside N(6')-acetyltransferase (plasmid)
WP_079849740	aminoglycoside 6'-acetyltransferase

Table S3: List of 43 sequences that convey resistance to *bla*.

NCBI accession number	Protein name
ABQ42698	AmpC beta-lactamase
WP_063857785	cephalosporin-hydrolyzing class C beta-lactamase ACT-8
AKN19343	blaCMY-16 (plasmid)
CQR83532	beta-lactamase/D-alanine carboxypeptidase
WP_032492458	CMY-1/MOX family class C beta-lactamase CMY-11
SLR23824	beta-lactamase
AFK78219	extended-spectrum beta-lactamase GES-11
AHD24679	beta-lactamase BEL-1 (plasmid)
AMS25623	carbapenem-hydrolyzing class A beta-lactamase KPC-34
ABF29644	beta-lactamase, partial (plasmid)
EJK91031	beta-lactamase
AAG45716	extended spectrum beta lactamase (plasmid)

WP_063860923	MULTISPECIES: class A broad-spectrum beta-lactamase OKP-B-5
AMM70781	TEM family class A beta-lactamase, partial
AKE33357	CTX-M1, partial
WP_014228247	MULTISPECIES: class A extended-spectrum beta-lactamase OXY-1-1
WP_049127974	OXY-2 family class A extended-spectrum beta-lactamase
ABQ10556	extended-spectrum beta-lactamase PER-1
ACG50807	extended-spectrum beta-lactamase PER-1, partial
ACG50811	extended-spectrum beta-lactamase PER-1, partial
CAA63714	extended-spectrum beta-lactamase
WP_063859341	RTG family carbenicillin-hydrolyzing class A beta-lactamase CARB-5
ACN89662	beta-lactamase CARB-4
KMX40042	beta-lactamase PSE-1
AMW97111	Beta-lactamase
SCY69726	Beta-lactamase OXA-1 precursor
WP_013279374	class D beta-lactamase
CHQ03353	Beta-lactamase OXA-10 precursor
CAP69660	OXA-129 beta-lactamase
YP_006953608	OXA-2 (plasmid)
ACC55538	Beta-lactamase class D
YP_006958782	OXA-48 (plasmid)
YP_001928076	OXA-9 (plasmid)
AJD77126	beta-lactamase Oxa9-like protein (plasmid)
EWE59955	beta-lactamase OXA-9
YP_008725242	metallo-beta-lactamse IMP-4 (plasmid)
AAK13430	metallo-beta-lactamase IMP-8 (plasmid)
WP_063860573	subclass B1 metallo-beta-lactamase IMP-11
ACT80138	metallo-beta-lactamase IMP-14a
ARJ98520	subclass B1 metallo-beta-lactamase
AAX76774	metallo-beta-lactamase SIM-1
ABS29633	metallo-beta lactamase protein
WP_013149463	VIM family subclass B1 metallo-beta-lactamase

Table S4: List of 28 sequences that convey resistance to *dfr*.

NCBI accession number	Protein name
YP_002527542	dihydrofolate reductase type I DhfrA1 (plasmid)
CAX63171	dihydrofolate reductase
KRR78083	hypothetical protein AR269_18705, partial
AAF27725	dihydrofolate reductase DHFRXVb
WP_063844288	trimethoprim-resistant repeat-containing dihydrofolate reductase DfrA1
AAA92749	dihydrofolate reductase (plasmid)
AAX18270	dihydrofolate reductase type IIIb (plasmid)
WP_063106511	hypothetical protein
YP_008060587	dihydrofolate reductase
WP_032165731	DfrA13/DfrA21 family trimethoprim-resistant dihydrofolate reductase
WP_012221074	trimethoprim-resistant dihydrofolate reductase DfrA12
YP_006953259	DfrA14, trimethoprim-insensitive class A dihydrofolate reductase (plasmid)
CUW83646	Dihydrofolate reductase type 5
AIU96905	dihydrofolate reductase (plasmid)
WP_015345005	dihydrofolate reductase
AQS26692	dihydrofolate reductase
ACJ22910	dihydrofolate reductase-like protein
CAD11597	dihydrofolate reductase
KTO00745	dihydrofolate reductase
AID93395	dihydrofolate reductase (plasmid)
APD70467	DfrA23
WP_074325914	trimethoprim-resistant dihydrofolate reductase DfrA24
CAL48457	putative trimethoprim resistance dihydrofolate reductase
AIX48195	dihydrofolate reductase
AAY33960	dihydrofolate reductase (plasmid)
YP_009182144	dihydrofolate reductase (plasmid)
ACR57831	dihydrofolate reductase type II
WP_063844479	trimethoprim-resistant dihydrofolate reductase DfrB6

Table S5: List of 64 essential gene sequences that do not convey resistance.

NCBI accession number	Protein name
CCZ50495	dNA primase
CCZ50068	dTDP-4-dehydrorhamnose 3 5-epimerase
CCZ50185	na+ dependent nucleoside transporter domain protein
CCZ50559	aspartate-semialdehyde dehydrogenase
CCZ50590	single-stranded DNA-binding protein
CCZ50619	hypoxanthine phosphoribosyltransferase
CCZ50687	serine acetyltransferase
CCZ50811	enoyl-[acyl-carrier-protein] reductase [NADH]
CCZ51255	dNA gyrase subunit B
CCZ51266	nucleoside diphosphate kinase
CCZ51280	dNA polymerase III subunit beta
CCZ51621	uDP-N-acetylglucosamine–N-acetylmuramyl-(pentapeptide) pyrophosphoryl-undecaprenol N-acetylglucosamine transferase
CCZ51503	malonyl CoA-acyl carrier protein transacylase
CCZ49657	dihydropteroate synthase
WP_024131855	long-chain-fatty-acid–CoA ligase FadD
ABX21148	hypothetical protein SARI_01246
ABX21165	hypothetical protein SARI_01263
ABX21677	hypothetical protein SARI_01791
ABX22149	hypothetical protein SARI_02286
WP_000121960	DNA polymerase III subunit gamma/tau
WP_012210538	undecaprenyldiphospho-muramoylpentapeptide beta-N-acetylglucosaminyltransferase
ABX23376	hypothetical protein SARI_03561
ABX23719	hypothetical protein SARI_03925
ABX23736	hypothetical protein SARI_03942
ABX24112	hypothetical protein SARI_04330
ABX24229	hypothetical protein SARI_04454
ABX20543	hypothetical protein SARI_00617
AKT91479	chromosomal replication initiator protein

AKT91578	DNA topoisomerase I
AKT92658	serine O-acetyltransferase
AKT91480	DNA polymerase III, beta subunit
AKT92884	cytidylate kinase
AKT92887	dihydropteroate synthase
AKT92937	enoyl-[acp] reductase
AKT93080	aspartate-semialdehyde dehydrogenase
AKT93081	DNA gyrase, subunit A
AKT93084	DNA polymerase III, gamma and tau subunits
AKT93257	nucleoside diphosphate kinase
AKT93280	DNA polymerase III, alpha subunit
AKT91859	malonyl-CoA-[acp] transacylase
AKT92225	primosomal protein N'
STP18534	nucleoside diphosphate kinase
AAN82658	Aspartate-semialdehyde dehydrogenase
WP_064221542	lipopolysaccharide core heptose(II) kinase RfaY
AAN83054	DNA gyrase subunit B
WP_000673456	MULTISPECIES: DNA polymerase III subunit beta
WP_016231270	chromosomal replication initiator protein DnaA
ABE08803	transport of nucleosides, permease protein
WP_060615308	DNA topoisomerase IV subunit B
WP_000918851	DNA primase
WP_001047338	MULTISPECIES: octaprenyl diphosphate synthase
WP_000383427	DNA helicase II
WP_000194413	cysteine hydrolase
ABE09848	multifunctional protein; 3-hydroxyacyl-CoA dehydrogenase
CDL60715	DNA polymerase III alpha subunit
CDL61206	Phosphopantothenoylcysteine decarboxylase / Phosphopantothenoylcysteine synthetase
CDL61311	3-oxoacyl-[acyl-carrier-protein] synthase, KASII
CDL61334	CDP-diacylglycerol–serine O-phosphatidyltransferase
CDL61377	DNA primase

CDL61843	dTDP-4-dehydrorhamnose 3,5-epimerase
CDL63342	Pyridoxine 5'-phosphate synthase
CDL64114	Phosphatidylglycerophosphatase A
CDL64552	UDP-N-acetylglucosamine–N-acetylmuramyl-(pentapeptide) pyrophosphoryl-undecaprenol N-acetylglucosamine transferase
CDL65005	3-oxoacyl-[acyl-carrier-protein] synthase, KASIII

Table S6: List of 7 histone acetyltransferases that do not convey resistance.

NCBI accession number	Protein name
OBL67881	histone acetyltransferase
ALX99713	histone acetyltransferase
KZO64708	histone acetyltransferase
OJR64716	histone acetyltransferase, partial
OCL31643	histone acetyltransferase
AJC06431	histone acetyltransferase
PAW16127	histone acetyltransferase

Unique protein names with NCBI accession numbers obtained from *Pseudomonas*, *Vibrio*, and *Enterobacter*

Table S7: List of 10 sequences that convey resistance to *aac*.

NCBI accession number	Protein name
CAA39184	aminoglycoside-(3)-N-acetyl-transferase isoenzyme III
AAN61405	aminoglycoside 3-N-acetyltransferase type II
CAD38269	gentamicin resistance protein, partial
AAB60000	aminoglycoside-(3)-acetyltransferase AacC-A1 or AacC1 or AAC-(3)-Ia (plasmid)
AAA25688	6'-N-acetyltransferase
AAA25680	aminoglycoside 6'-N-acetyltransferase
AAL82588	aminoglycoside 3-N-acetyltransferase/aminoglycoside 6'-N-acetyltransferase fusion protein
CAE48335	AAC(6')-30/AAC(6')-Ib'

AAA16194	AAC 3-VI protein
CAA39038	AG-6'-acetyltransferase

Table S8: List of 43 sequences that convey resistance to *bla*.

NCBI accession number	Protein name
AAG07497	beta-lactamase precursor
ABR85124	beta-lactamase
AAY93315	beta-lactamase
AAK18183	beta-lactamase IBC-2
ABY91240	beta-lactamase KPC-5
AAC64364	beta-lactamase CEF-1 precursor
AAN61404	extended-spectrum beta-lactamase TEM-21
ABC54728	beta-lactamase CTX-M-2 precursor (plasmid)
AAW62294	PER-1
AAC27624	beta-lactamase (plasmid)
AAC09012	CARB-4 precursor (plasmid)
AAM74565	carbenicillin-hydrolyzing beta-lactamase
BAE71359	beta-lactamase
CAI46978	Class-A beta-lactamase (plasmid)
AAK14791	group 2d beta-lactamase NPS-1 (plasmid)
AAK52604	OXA-31 (plasmid)
ACI29753	GcuF1/OXA-28 fusion protein
AAB05874	beta-lactamase OXA-15
AAN63499	OXA-like protein
AAM08183	beta-lactamase OXA-5
ABA54978	OXA-9
CAF05908	GIM-1 protein
AAK12087	metallo-beta-lactamase
AAK59385	IMP-9 metallo-beta-lactamase (plasmid)
AAT49068	IMP-14
ABC88434	metallo-beta-lactamase IMP-22
ACL31199	IMP-13

CAD12765	metallo beta-lactamase
CAD61201	metallo-b-lactamase
CAE46566	metallo-beta-lactamase
AAM11666	AmpC
AAO42602	beta-lactamase Mir2
AAP93849	beta-lactamase, partial
ABF29651	class C beta-lactamase (plasmid)
ABF29654	class C beta-lactamase (plasmid)
ABF29657	class C beta-lactamase (plasmid)
BAA07922	class C beta-lactamase precursor
CAG28257	beta-lactamase class C
AAK31368	extended-spectrum beta-lactamase precursor
AAT70415	CTX-M-37 beta-lactamase (plasmid)
AAZ30046	CTX-M-9 (plasmid)
ABM73648	KLUC-2 (plasmid)
AAA93461	IMI-1

Table S9: List of 8 sequences that convey resistance to *dfr*.

NCBI accession number	Protein name
ABA38890	dihydrofolate reductase type I
BAD51733	dihydrofolate reductase
ABV21791	dihydrofolate reductase
ABG91835	DHFRXVII
EEO11750	dihydrofolate reductase
CAQ52800	DHFR2 protein
AAT49071	dihydrofolate reductase
ACO54029	DfrA19 (plasmid)

Table S10: List of 25 essential gene sequences that do not convey resistance.

NCBI accession number	Protein name
WP_022960967	pyruvate dehydrogenase (acetyl-transferring) E1 component subunit alpha

WP_022961210	hypoxanthine-guanine phosphoribosyltransferase
WP_022961823	serine O-acetyltransferase
WP_022961824	nucleoside-diphosphate kinase
WP_028614653	undecaprenyldiphospho-muramoylpentapeptide beta-N-acetylglucosaminyltransferase
WP_040640850	d)CMP kinase
WP_022962405	[acyl-carrier-protein] S-malonyltransferase
WP_022962412	DNA polymerase III subunit delta'
WP_022963226	DNA polymerase III subunit gamma/tau
WP_021018876	MULTISPECIES: 1,4-dihydroxy-2-naphthoyl-CoA synthase
WP_021019516	DNA polymerase III subunit gamma/tau
WP_021019431	DNA polymerase III subunit delta
WP_027694166	quinolinate synthase NadA
ASA54362	DNA gyrase subunit A
WP_021020447	CDP-diacylglycerol–glycerol-3-phosphate 3-phosphatidyltransferase
WP_021020875	permease
WP_021020932	(2E,6E)-farnesyl diphosphate synthase
WP_021020950	MetQ/NlpA family lipoprotein
EGK62537	APC family amino acid transporter
EGK60566	CaCA family calcium (Ca2+):cation antiporter
EGK58767	chloride channel (ClC) family chloride transporter
WP_013098318	ribonuclease III
WP_010426391	lipopolysaccharide heptosyltransferase RfaC
WP_010426438	MULTISPECIES: guanylate kinase
KDF55569	chromosomal replication initiator protein dnaA

Table S11: List of 8 histone acetyltransferases that do not convey resistance.

NCBI accession number	Protein name
GBH15333	histone acetyltransferase
GBH08811	histone acetyltransferase
PYD93376	histone acetyltransferase
PYD91560	histone acetyltransferase

PYD90245	histone acetyltransferase
KTB87679	histone acetyltransferase
RFD66926	histone acetyltransferase
KKP22059	histone acetyltransferase
